# Inhibitory effect of esculentoside A on tumour necrosis factor α production by human monocytes

**DOI:** 10.1155/S0962935196000427

**Published:** 1996-09

**Authors:** H-B. Wang, J. Fang, Q-Y. Zheng

**Affiliations:** 1 Department of Pharmacology College of Pharmacy Second Military Medical University Shanghai 200433 China

## Abstract

Esculentoside A (EsA) is a saponin isolated from the roots of *Phytolacca esculenta*. Previous experiments have shown that it has strong anti-inflammatory effects. Tumour necrosis factor (TNF) is a very important inflammatory mediator. It is known that there are two types of TNF—TNFα is from macrophages/monocytes and TNFβ is from activated lymphocytes. In order to study the mechanism of the anti-inflammatory effect of EsA, it was determined whether TNFα production from human peripheral monocytes was altered by EsA under lipopolysaccharide (LPS)-stimulated conditions. EsA was found to decrease TNFα production in a dose-dependent manner at concentrations higher than 1 μmol/l EsA. Recent studies have shown that EsA has a curative effect on chocolate cyst and other inflammatory diseases. Our previous studies have shown that EsA could reduce the release of platelet activating factor (PAF) from rat macrophages, and inhibit interleukin-1 and interleukin-6 production from routine macrophages. The reducing effects of EsA on the release of TNFα, IL-1, IL-6 and PAF may explain its anti-inflammatory effect.


Mediators of Inflammation 5, 292–294 (1996)

### Inhibitory effect of *esculentoside* A on tumour necrosis factor α production by human monocytes

H-B. Wang,[CA] J. Fang and Q-Y. Zheng

Department of Pharmacology, College of Pharmacy, Second Military Medical University, Shanghai 200433, People's Republic of China

[CA]Corresponding Author

***Esculentoside* A (EsA) is a saponin isolated from the roots of *Phytolacca esculenta*. Previous experiments have shown that it has strong anti-inflammatory effects. Tumour necrosis factor (TNF) is a very important inflammatory mediator. It is known that there are two types of TNF—TNFα is from macrophages/monocytes and TNFβ is from activated lymphocytes. In order to study the mechanism of the anti-inflammatory effect of EsA, it was determined whether TNFα production from human peripheral monocytes was altered by EsA under lipopolysaccharide (LPS)-stimulated conditions. EsA was found to decrease TNFα production in a dose-dependent manner at concentrations higher than 1 μmol/l EsA. Recent studies have shown that EsA has a curative effect on chocolate cyst and other inflammatory diseases. Our previous studies have shown that EsA could reduce the release of platelet activating factor (PAF) from rat macrophages, and inhibit interleukin-1 and interleukin-6 production from murine macrophages. The reducing effects of EsA on the release of TNFα, IL-1, IL-6 and PAF may explain its anti-inflammatory effect.**

**Key words:** *Esculentoside* A, Human peripheral monocyte, Tumour necrosis factor α

### Introduction

*Esculentoside* A (EsA) is a saponin isolated from the root of *Phytolacca esculenta*, and is identified as: 3-O-[β-D-glucopyranosyl-(1-4)-β-D-xylo-pyranosyl] phytolaccagenin. The structure of this compound is shown in Fig. 1. Experiments have shown that it has strong anti-inflammatory effects.[1] It is now known that tumour necrosis factor (TNF) possess a number of properties of inflammatory response.[2,3] Platelet activating factor (PAF), interleukin-1 and interleukin-6 are also inflammatory mediators.[3] It has been shown that EsA inhibited the production of PAF by $A_{23187}$ stimulated rat macrophages,[4,5] and the production of TNFα, IL-1, IL-6 of LPS stimulated murine peritoneal macrophages.[6,8] The aim of this work was to evaluate the effects of EsA on the production of TNFα by LPS stimulated human peripheral monocytes.

### Materials and Methods

*Reagent:* RPMI-1640, lipopolysaccharide (*Escherichia coli* 055:B5) and calcimycin ($A_{23187}$) were purchased from Sigma (USA).

*Human peripheral monocyte preparation:* Human monocytes were isolated by a combination of Ficoll-Hypaque gradient centrifugation and centrifugal elutriation. This procedure yielded populations of monocytes with greater than 95% purity. The cells were washed three times in RPMI-1640 and suspended at 1 × $10^6$ cells per 1 ml in freshly prepared standard culture medium. The cells were plated at 1 ml of cells per well.

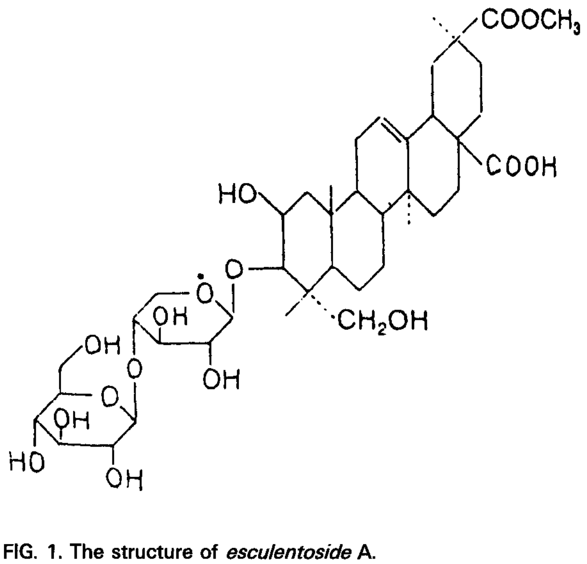

FIG. 1. The structure of *esculentoside* A.

© 1996 Rapid Science Publishers

*TNFα production:* Two hours later, the medium containing non-adherent cells was decanted and the non-adherent cells in supernatant were counted to measure the adherence ratio. TNFα activity was expressed as U/$10^6$ monocytes. The adherent cells were rinsed twice with Hanks's solution. Then the adherent cells were incubated with $A_{23187}$ (1 μmol/l) for 6 h. After incubation, the medium was discarded, and the cells were washed three times with RPMI-1640 to remove $A_{23187}$. Fresh culture medium without serum was added to every well with lipopolysaccharide (LPS, 10 μg/ml) in the presence or absence of EsA. The cultures were incubated in a humidified atmosphere of 5% $CO_2$ and at 37°C for another 6 h. The supernatants were harvested and centrifuged. The cell-free supernatants were collected, dialysed in phosphate-buffer solution for 24 h, and stored at −20°C prior to activity assay.

*TNFα production assay:* TNFα assay was performed essentially as described by Kunkel *et al.*[5] with minor modifications. Briefly, $L_{929}$ cells (50 000/well) were dispensed into 96-well flat-bottomed microtitre plates in a volume of 0.1 ml/well. The following day, the cells were incubated for 18 h in the presence of 1 μg of actinomycin D and serial 1:2 dilutions of test sample. Media were then decanted, and the remaining cells in each well were stained with crystal violet for about 15 min, washed with tap water, and dried at 40°C. Absorbance of the cells in each well was read using a microenzyme linked immunosorbent autoreader. Units of TNFα activity were defined as described by Kunkel *et al.*[5]

*Cell viability:* The trypan blue exclusion test was performed after 6 h incubation with or without EsA. Cell viability was also determined by measuring lactate dehydrogenase (LDH) activity in the cell-free supernatants according to the method of Beutle.[9]

*Statistics:* Each experiment was carried out at least three times. Values were expressed as the mean ± standard error. Variation between parallel experiments was less than 30%. Probability values for statistical differences were determined by Student's *t*-test and *p* values of less than 0.05 were considered significant.

### Results

*Effects of EsA on production of TNFα from human monocytes:* TNFα in the supernatant after being dialysed was assessed by the killing of $L_{929}$ cells. One unit of TNFα was defined as the reciprocal of the dilution of a preparation that results in 50% survival of the cells. The results are presented in Table 1. It is observed that the decrease of TNFα production was significant at the concentrations of 1 and 10 μmol/l EsA.

**Table 1.** Effect of *esculentoside* A on tumour necrosis factor α production from $10^6$ human peripheral monocyte stimulated by LPS 10 μg/ml, mean ± SD, *n* = 3 samples

| | TNFα activity (U/$10^6$) |
|---|---|
| LPS | 51.4 ± 4.6 |
| LPS+EsA (μmol/l) | |
| 0.01 | 55.0 ± 13.5 |
| 0.1 | 45.2 ± 8.9 |
| 1.0 | 36.5 ± 3.6* |
| 10.0 | 27.1 ± 6.1* |

*$p < 0.05$ versus control group.

*Effects of EsA on the kinetics of TNFα production from human monocytes:* Monocytes were cultured in LPS (10 μg/ml) with or without EsA, and the supernatants were harvested at 2 h intervals. TNFα activity can be detected after 2 h exposure to LPS. Figure 2 shows that TNFα production by human monocytes were inhibited in a time dependent manner by EsA at a concentration of 5 μmol/l.

*Effects of EsA on cellular activity:* In order to eliminate the possibility that EsA was toxic for the cells tested, cell viability was monitored by measuring LDH activity in the supernatant and by the trypan blue exclusion test performed at the end of the macrophage culture. Results of the

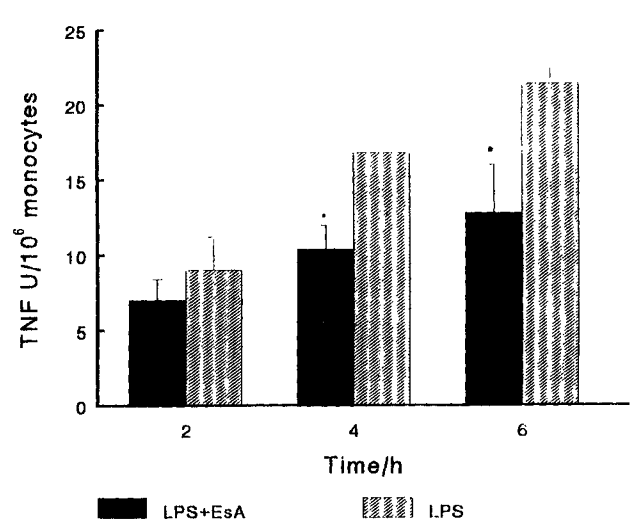

FIG. 2. Effect of EsA on the kinetics of TNFα production in supernatants from human peripheral monocytes stimulated with 10 μg/ml LPS for 2, 4 and 6 h. The concentration of EsA was 5 μmol/l, *n* = 3, mean ± SD, *$p < 0.05$ versus LPS.

**Table 2.** LDH activities in supernatants and cell viability ratio of human peripheral monocytes treated with or without *esculentoside* A. $n = 3$ samples, mean $\pm$ SD

| | LDH activity (U/ml) | Cell viability ratio (%) |
|---|---|---|
| Control | $4.35 \pm 0.17$ | >99 |
| EsA (μmol/l) | | |
| 0.01 | $4.47 \pm 0.19$ | >99 |
| 0.1 | $4.29 \pm 0.14$ | >99 |
| 1.0 | $4.55 \pm 0.16$ | >99 |
| 10.0 | $4.44 \pm 0.35$ | >99 |

LDH in supernatant of adherent macrophages, cultured for 6 h with various concentrations of EsA, was not different from that of the control, as is shown in Table 2. Cell viability was confirmed by the trypan blue exclusion test. The cell viability ratio in test samples was more than 99% (Table 2). The result was the same as previously reported.[10]

### Discussion

In this study, EsA induced a dose-dependent decrease in the TNFα concentrations measured in the supernatant of LPS-stimulated human monocytes. The kinetics of TNFα production were also changed. TNF was initially identified as a factor that appeared in the circulation of animals following the injection of endotoxins. TNFα is a product of stimulated monocytes and macrophages, but it is also produced by keratinocytes.[6] In addition to the cytotoxic activities of TNFα in some types of transformed cells, recent data have shown that TNFα mediated stimulation of collagenase synthesis and prostaglandin $E_2$ ($PGE_2$) production by synovial cells,[7] and stimulated bone resorption and inhibition,[8] suggesting that TNFα might be an important mediator of inflammation. Platelet activating factor (PAF) is a mediator of anaphylaxis and inflammation; it plays an important role in inflammation, and there is cooperation between PAF and TNFα in inflammatory reactions.[11] It has been found that EsA reduced release of PAF from rat macrophages. A previous study also showed that EsA can inhibit the inflammatory reaction induced by carrageenan.[1] Recent clinical trials showed that a Chinese herb containing EsA had a significant curative effect on chocolate cyst. Our recent test showed that EsA could significantly inhibit IL-1 and IL-6 production from murine macrophages.[12] Thus, together with the present investigation it is suggested that the anti-inflammatory effects of EsA might be due to its reducing effects on the release of TNFα, PAF, IL-1, IL-6 and other inflammatory mediators.

### References

1. Zheng QY, Mai K, Pan XF. Antiinflammatory effects of *esculentoside* A. *Chin J Pharmacol Toxicol* 1992; **6:** 221–224.
2. Dinarello CA, Mier JW. Lymphokines. *New Engl J Med* 1987; **317:** 940–945.
3. Maestre P, Zarco C, Guerrero CG. Cooperation between tumor necrosis factor (TNF) and platelet-activating factor (PAF) in the inflammatory response. *J Lip Med* 1990; **2:** s151–s159.
4. Fang J, Zheng QY. Inhibitory effects of *esculentoside* A on platelet activating factor released from calcimycin induced rat peritoneal macrophages. *Acta Pharmaceutica Sin* 1991; **26:** 721–724.
5. Kunkel SL, Spengler M, May MA, Spengler R, Larrick J, Remick D. Prostaglandin E2 regulates macrophages-derived tumor necrosis factor gene expression. *J Biochem* 1988; **263:** 5380–5384.
6. Dinarello CA. *Cytokines: Interleukin-1 and Tumor Necrosis Factor (Cachectin).* New York: Raven Press, 1988: 54.
7. Dayer JM, Beutler B, Ceram A. Cachectin/tumor necrosis factor stimulates collagenase and prostaglandin $E_2$ production by human synovial cells and dermal fibroblast. *J Exp Med* 1985; **162:** 2143–2168.
8. Bertolini DR, Nedwin GE, Bringman TS, Smith DD, Mundy GR. Stimulation of bone resorption and inhibition of bone formation *in vitro* by human tumor necrosis factor. *Nature* 1986; **319:** 516–518.
9. Beutle E. *Lactate Dehydrogenase.* 2nd edn. New York: Grune & Stratton, 1975, 63–64.
10. Fang J, Zheng QY, Wang HB, Yi YH. Effects of *esculentoside* A on tumor necrosis factor production by mice peritoneal macrophage. *Mediators of Inflammation* 1992; **1:** 375–377.
11. Braquet P, Braquet MP, Bourgaain QH, Bussolin F, Hosford D. PAF/cytokin auto-generated feedback networks in microvascular immune injury: consequences in shock, ischemia and graft rejection. *J Lip Med* 1989; **1:** 75–112.
12. Ju DW, Zheng QY, Wang HB, Fang J. Effect of *esculentoside* A on immune function in mice. *Acta Pharmaceutica Sin* 1994; **29:** 252–255.

**Received 10 June 1996; accepted in revised form 17 July 1996**
